# An assessment of adverse drug reactions among HIV positive patients receiving antiretroviral treatment in South Africa

**DOI:** 10.1186/s12981-015-0044-0

**Published:** 2015-03-05

**Authors:** Lieketseng J Masenyetse, Samuel OM Manda, Henry G Mwambi

**Affiliations:** Biostatistics Unit, South African Medical Research Council, Pretoria, South Africa; School of Mathematics, Statistics and Computer Science, University of KwaZulu-Natal, Pietermaritzburg, South Africa

**Keywords:** Antiretroviral treatment, Adverse drug reactions, Recurrent events

## Abstract

**Background:**

Antiretroviral treatment (ART) has been effective in reducing HIV/AIDS related morbidity and mortality. However, the use and uptake of ART has resulted in adverse reactions, due mainly to the medicine’s toxicity and interactions with other medicines. The timing of adverse drug reactions (ADRs) among these patients is a critical public health issue for antiretroviral (ARV) treatment adherence and retention. Reliable monitoring of HIV patients on ART is through a structured pharmacovigilance surveillance system. However, recurrent nature of these data pose challenges in their analyses. This study aimed at modelling the timing of ADR events in HIV patients on ART using correlated time-to-event models.

**Methods:**

The data concern 590 HIV patients registered onto the Medunsa National ARV Pharmacovigilance Surveillance System within 6 months of ART initiation between February 2007 and July 2011. Recurrent times of ADRs and baseline characteristics: patient gender, and age, ART regimen, clinic and initiation period were extracted from the data. The recurrent ADR events data were modelled using both shared frailty and marginal models on the five patients’ characteristics as covariates.

**Results:**

Out of 590 patients, 67% were female, 68% started on regimen: Stavudine, Lamivudine and Efavirenz; 37% had experienced at least one ADR and 67% started ART in 2009–2011. Age (p-value = 0.0210), clinic (p-value < 0.0001) and period of ART initiation (p-value = 0.0002) were significantly associated with timing of first ADR. There was a significantly higher rates of ADR recurrences in patients aged 38–44 years [HR = 2.45; 95% CI = (1.47; 4.10)] vs. 30 years and less, patients taking regimen: Zidovudine, Lamivudine and Nevarapine) vs. regimen: Stavudine, Lamivudine and Efavirenz [HR = 2.09; 95% CI = (1.35; 3.22)], while the rate was lower among those who started ART in 2009–2011 vs. those who initiated in 2007–2008 [HR = 0.55; 95% CI = (0.40; 0.76)].

**Conclusion:**

More realistic time-to-event models for recurrent events data have been used to analyse timing of ADR events in HIV patients taking ARV treatment. Age, antiretroviral regimen type and period of initiation of ART were associated with the timing of HIV/AIDS drug related adverse reactions regardless of the analysis model used. This study has public health policy implications in addressing the added morbidity among HIV patients taking ARV treatment in the context of universal scaling up of ARV treatment.

## Background

The Human Immunodeficiency Virus (HIV) has changed from life threatening to chronic condition due to the almost universal use and accessibility of antiretroviral treatment (ART) among HIV patients [1]. Antiretroviral (ARV) treatment works by providing suppression of viral load and restoring the immune system. It is estimated that out of the 35.3 million people living with HIV worldwide, 10.6 million were receiving ART in 2012 [2]. Nearly, 6.6 million HIV/AIDS related deaths worldwide have been prevented as a result of ART [2]. Despite these gains, adverse reactions to these medicines remain a significant public health concern and may compromise the effectiveness of the ART programmes [3,4].

The risk of adverse drug reactions (ADRs) arises because of the effect of the disease on the immune systems and the safety profiles of the complex ART drugs [3]. There are a number of ADRs related to ART that have been documented, and may be mild to severe; and short to long term depending on the environment [1,5-12]. ADRs in developing countries may differ from those in developed countries because of high prevalence of conditions such as malnutrition, tuberculosis and patients presenting with advanced HIV disease [13]. For instance, it has been found that in Africa, neuropathy, neutropenia and lipodystrophy are the predominant ADRs [14]. Short term ADRs are a potential threat to successful initiation and adherence to ART [15]. The timing of ADRs may also depend on the type of drugs. Studies have shown that patients on Efavirenz, Lamivudine and Zidivudine or Indinavir, Zidovudine and Lamivudine may present with ADRs within the first 12 or 24 weeks, respectively [16,17]. ADRs may be common or specific to class of drugs [1,8,15]. Drugs classified as non-nucleoside reverse transcriptase inhibitors (NNRTIs) which include Efavirenz (EFZ) and Nevirapine (NVP) are known to cause rashes and hepatotoxicity. On the other hand drugs classified as nucleoside reverse transcriptase inhibitors (NRTIs) including Zidovudine (AZT) and Stavudine (d4T) are known to cause anemia, nausea, rashes, lipoatrophy and lactic acidosis [1].

Apart from ADR depending on the environment and the type of ART regimen, a number of other risk factors have been identified, that include patient age, gender, duration on treatment, disease biomarkers such as CD4 count and viral load and body mass index (BMI) [7,10,18,19]. These risk factors have been found to interact with type of ADR. For instance females are more likely to develop rashes and hepatotoxicity [7,18]; and patients aged 40 years and above are at a higher risk of developing peripheral neuropathy when taking d4T [10]. The longer a patient is on ART the less likely they would experience ADRs; possibly as a result of stability in ARV regimen, coming after many changes and eventually settling on an acceptable regimen [19].

Monitoring safety and toxicity related to ART remains a challenge facing the public health sector. Monitoring is usually done using spontaneous surveillance of HIV patients on treatment. Spontaneous reporting of ADRs is a very inefficient system in detecting drug-related conditions, leading to underestimation of the burden due to ADRs [3,20,21]. Thus, more systematic and more robust surveillance methods including structured surveillance pharmacovigilance systems, which assesse and monitor safety profile and impact of antiretroviral medicines have been advocated [4]. Structured surveillance tracks HIV positive patients who are on ART to assess drug related morbidity and mortality over time. South Africa, a country heavily hit by the HIV epidemic, uses spontaneous surveillance of HIV patients on ART to assess ART-related adverse effects. Though these data are routinely available, the coverage of important patient data may not be adequate. Thus, for the purposes of this study, data from a structured surveillance system in South Africa are used.

The adverse drug reaction events in patients often are of recurrent nature, such that the repetitions tend to cluster more in some patients than in others. Analyses of these data are complicated due to the fact that independence between the recurrent event times cannot be assumed in a subject. In medical studies, time-to-event models have been developed to account for possible dependence between recurrent events data [22,23]. The aim of this paper was to provide a unified analysis of recurrent ADR events data from a structured antiretroviral pharmacovigilance surveillance system. The authors are not aware of any such study that has comprehensively analysed data from this kind of structured surveillance system for HIV positive patients. A previous study investigated ADRs in a sample of adult inpatients at a local hospital in South Africa, and compared the distribution between HIV and non-HIV patients [21]. This present study is very specific and unique by assessing ARV-related ADRs in a cohort of only HIV patients in a country with one of the highest HIV burden of the disease and with over 75% of the 6.1 million HIV patients on free ART [24].

## Methods

### Data

Data are from the Medical University of South Africa (MEDUNSA) National ARV Pharmacovigilance Centre, which is based at the University of Limpopo MEDUNSA campus. Recruitment of patients started in January 2007 from four clinics, two in Gauteng, one in Limpopo and one in Mpumalanga provinces. Patients were followed up to obtain information on the ADRs they were experiencing and these data were collected during patients’ clinic visitations. Other information contained in the data were the type of regimens patients were taking together with the dates when they switched or collected the same regimens. A descriptive of the data generated by the system can be found in Dube et al. [9].

The study included HIV positive patients aged 15 years and above, receiving ART in the South Africa public health sector [9]. Patients who met the inclusion criteria were recruited when visiting public health sector clinics using systematic random sampling. Patients who agreed to participate in the study were asked to sign consent forms. Patients who were antiretroviral naïve or on ART at private clinics were excluded from the study. Further details about the study can be found in Dube et al. [9]. For purposes of this paper, data were restricted only to 590 patients enrolled within 6 months of their ART initiation between January, 2007 and August, 2011. The covariates considered in this analysis were age, gender, clinics and antiretroviral medication patients are taking.

### Statistical analysis

In previous analyses of these types of data, standard logistic and survival models, assuming ADR events as independent, were fitted [20,21]. For this study ADR recurrent events in a patient could not be taken to be independent of each other: some patients could have been more prone to experience ADRs than others. Thus analyses of these data must take into account; possible dependence in the occurrence of ADRs. This study used more robust modelling techniques based on time-to-event models for correlated survival data. In particular, the marginal and shared frailty models were used to model the recurring ADR events data in order to take account of possible correlation between the data [22,23].

There are some analytical assumptions differentiating the two proposed correlated survival models. The marginal model fits separate failure times using survival models, while the dependence structure unspecified but inflates the estimated variance-covariance parameters of regression coefficients [23]. Under the shared frailty model subject-specific random effects, which capture dependence in the failure times within a subject, are assumed to be time constant and independent and identically distributed from a known distribution function. This can be relaxed to have the subject-specific random effects to be time-varying as in Manda and Renate [22] and to be distributed nonparametrically as in Manda [25]. The fitting of the models were implemented in STATA version 12 as described in Cleves and StataCorp [26].

## Results

Descriptive analyses using summary statistics was undertaken. These are shown in Table 1 for patients demographic and baseline characteristics. A majority of the patients were females accounting for 67% of the sample. Majority of the patients were from clinic A and clinic C accounting for about 86% of the sample, with 67% of the patients having started ART from 2009. Overall 217 (37%) patients out of 590 experienced at least one ADR, most of them being females (72%), in Clinic C (75%), were on regimen 1(a) (66%) at the time of reporting these ADRs and had started ART from 2009. Of the 217 patients who had experienced at least one ADR, 61 (28%) patients had ADRs for the second time. Among patients experiencing a second ADR, a majority were females (74%), were still in regimen 1a (62%), were in the age group of 38 to 44 years (39%) and had started ART before 2009 (66%).

**Table 1:** **Patients demographic and baseline characteristics, HIV Patients on ART, South Africa 2007-2012**

**Characteristics**	**Total (%)**	**Patients with adverse drug effects (%)**		
		**0 ADR → 1** ^**st**^ **ADR**	**1** ^**st**^ **ADR → 2** ^**nd**^ **ADR**	**2** ^**st**^ **ADR → 3** ^**nd**^ **ADR**
Overall	590 (100)	217 (100)	61 (100)	24 (100)
**Gender**				
Male	192 (33)	61 (28)	16 (26)	5 (21)
Female	398 (67)	156 (72)	45 (74)	19 (79)
**Age at clinic visit**				
30 and less	87 (15)	22 (10)	2 (3)	0 (0)
31 – 37	183 (31)	64 (29)	14 (23)	5 (21)
38 – 44	154 (26)	70 (32)	24 (39)	10 (42)
45+	166 (28)	61 (28)	21 (34)	9 (37)
**Clinic**				
Clinic A	188 (32)	32 (15)	5 (8)	1(4)
Clinic B	31 (5)	4 (2)	0 (0)	0 (0)
Clinic C	318 (54)	162 (75)	48 (79)	19 (79)
Clinic D	53 (9)	19 (9)	8 (13)	4 (17)
**ART regimens**				
1a (d4T, 3TC, EFZ)*	402 (68)	144 (66)	38 (62)	12 (50)
1b (d4T, 3TC, NVP)*	67 (11)	20 (9)	3 (5)	1(4)
1c (AZT, 3TC, EFZ)*	46 (8)	25 (12)	12 (20)	4 (17)
Others	75 (13)	28 (13)	8 (13)	7 (29)
**Year started ART**				
Before 2009	194 (33)	94 (43)	40 (66)	21 (88)
2009 and after	396 (67)	123 (57)	21 (34)	3 (13)

*d4T = Stavudine; 3TC = Lamivudine; EFZ = Efavirenz; NVP = Nevarapine; AZT = Zidovudine.

**Figure 1:**
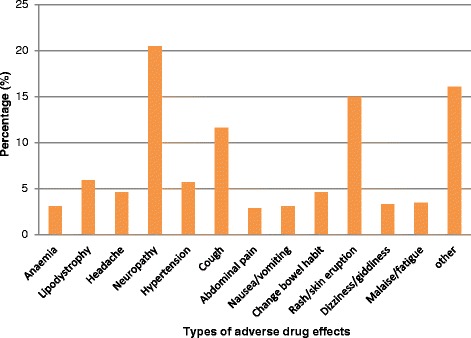
**Distribution of adverse drug reactions among HIV patients on ART, South Africa 2007–2012.**

Distribution of the observed ADRs is shown in Figure 1. There was a total of 454 ADRs for all the 217 patients who had experienced at least one ADR. Neuropathy accounted for 20% of all ADRs followed by rash/skin eruptions at 15%. Cough was also prevalent accounting for 12% of the ADRs. Other ADRs accounting for 16% of the total included those with proportions less than 2%, such as acidosis, dermatitis, oedema and hypothyroidism.

**Figure 2:**
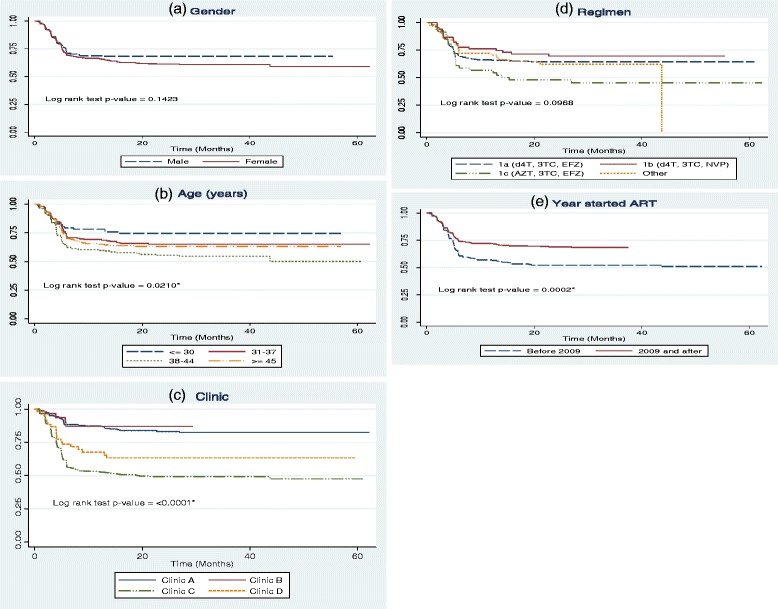
**Kaplan Meier curves for time to first ADRs among HIV patients on ART, South Africa 2007–2012 classified by (a) gender, (b) age, (c) clinic (d) regimen and (e) Year started ART. HIV.**

In order to investigate associations between the different covariates and timing of ADRs, Kaplan-Meier survival curves were used to describe differences in the survival rates. The log-rank test was used for testing equality of the curves between categories of the respective covariates on timing to first ADR event. The survival curves with the log-rank test are shown in Figures 2a-2e. Males had better survival of not experiencing ADRs compared to females, though the association was not significant (log rank test = 0.1423). Age had a significant effect on timing of experiencing first ADRs ( log rank test = 0.0210), with patients aged less than 30 years having lower ADR rates compared to those in older age groups. Clinic and year patients initiated ART also had significant effects on the timing of experiencing ADRs (p-value ≤ 0.05) the first time.

The multivariate analyses included all the covariates regardless of significance of associations from the univariate log-rank test in the preceding paragraph. In terms of modelling specific transitions between (from baseline to first, from first to second, from second to third and so forth) ADRs only the two transitions (baseline to first and first to second timings) were used. The later transitions had very few events of experiencing ADRs. We intially took these as two independent time-to-event processes, and thus can be modelled using standard proportional hazards models. The adjusted results from fitting these are shown in columns two and three of Table 2 on the hazard ratio (HR) scale. Using of separate transitions as independent was corrected by using all ADR events in a subject. These were fitted using both the marginal and frailty models as described in the Methods section. The adjusted results of these later two models are presented in the last two columns of Table 2, also on the HR scale.

**Table 2:** **Adjusted Hazard Ratios (HR) of various characteristics on the occurrence of ADRs, HIV Patients on ART, South Africa 2007-2012**

	**1** ^**st**^ **ADR**	**1** ^**st**^ **ADR → 2** ^**nd**^ **ADR**	**Marginal model**	**Frailty model**
**Characteristic**	**HR (95% CI)**	**HR (95% CI)**	**HR (95% CI)**	**HR (95% CI)**
**Gender**				
Male	1	1	1	1
Female	1.13 (0.82, 1.55)	1.32 (0.72, 2.42)	1.23 (0.88, 1.72)	1.23 (0.94, 1.62)
**Age**				
30 and less	1	1	1	1
31 – 37	1.33 (0.81, 2.19)	2.41 (0.54, 10.69)	1.59 (0.95, 2.66)	1.51 (0.94, 2.43)
38 – 44	1.83 (1.12, 3.01)^*^	3.19 (0.73, 13.93)	2.45 (1.47, 4.10)^*^	2.07 (1.29, 3.32)^*^
45+	1.43 (0.85, 2.42)	3.23 (0.73, 14.29)	2.02 (1.16, 3.51)^*^	1.80 (1.11, 2.93)^*^
**Clinic**				
Clinic A	1	1	1	1
Clinic B	0.77 (0.27, 2.20)	-	0.73 (0.25, 2.11)	0.78 (0.28, 2.22)
Clinic C	3.74 (2.54, 5.50)^*^	2.36 (0.89, 6.22)	4.03 (2.75, 5.91)^*^	3.20 (2.24, 4.57)^*^
Clinic D	1.99 (1.10, 3.58)^*^	2.76 (0.85, 8.99)	2.14 (1.12, 4.09)^*^	1.96 (1.18, 3.25)^*^
**ART regimens**				
1a (d4T, 3TC, EFZ)	1	1	1	1
1b (d4T, 3TC, NVP)	1.01 (0.61, 1.66)	0.65 (0.19, 2.21)	1.08 (0.65, 1.80)	0.99 (0.64, 1.55)
1c (AZT, 3TC, EFZ)	1.86 (1.20, 2.87)^*^	1.83 (0.91, 3.69)	2.09 (1.35, 3.22)^*^	1.83 (1.27, 2.62)^*^
Others	1.00 (0.65, 1.54)	1.18 (0.50; 2.82)	1.35 (0.90, 2.01)	1.43 (0.99, 2.04)
**Year started ART**				
Before 2009	1	1	1	1
2009 and after	0.78 (0.58, 1.06)	0.41 (0.23, 0.74)^*^	0.55 (0.40, 0.76)^*^	0.90 (0.70, 1.16)

*Statistically significant at p-value ≤ 0.05.

The rate of experiencing first and second ADRs were higher for females compared to males though not significant, [Hazard ratio = 1.13; 95% Confidence Interval = (0.82; 1.55)] and [HR = 1.32; 95% CI = (0.72; 2.42)] respectively. Older age groups had higher rate of experiencing ADR compared to the younger age group of 30 years and less, with significant risks being for patients in aged 38 – 44 years [HR = 1.83; 95% CI = (1.12; 3.01)]. Patients who were in regimen 1(c) had significantly higher risks of having an ADR compared to those in regimen 1(a) [HR = 1.86; 95% CI = (1.20; 2.87)]. Patients on regimen 1b had a lesser rate of experiencing recurrent ADRs compared to those in regimen 1a though this was not significant [HR = 0.65; 95% CI = (0.19; 2.21)].

The marginal model results indicate that patients in age 38 – 44 years and from 45 years had significantly higher rate of recurrence of ADRs compared to those 30 years and younger [HR = 2.45; 95% CI = (1.47; 4.10)] and [HR = 2.02; 95% CI = (1.16; 3.51)] respectively. Patients on regimen 1(c) had significantly higher hazards of recurrence of ADRs compared to patients on regimen 1(a) [HR = 2.09; 95% CI = (1.35; 3.22)]. Patients who initiated ART from 2009 had significantly lower hazards of recurrence of ADRs compared to patients who initiated ART before 2009 [HR = 0.55; 95% CI = (0.40; 0.76)].

Frailty model results show that 31 years and older patients experienced higher rates of ADRs compared to those aged 30 years and less. Patients who were initiated ART from 2009 had significantly lower risks of recurrence of ADRs compared to patients who were initiated before 2009 [HR = 0.90; 95% CI = (0.70; 1.16)], though this finding was not significant. Patients attending clinic C and D had significantly higher rate of recurrence of ADRs compared to patients in clinic A [HR = 2.07; 95% CI = (1.29; 3.32)] and [HR = 1.80; 95% CI = (1.11; 2.93)] respectively.

Timing of first ADR was also analysed using logistic regression where the outcome was categorized as 1 if a patient had an ADR and 0 otherwise. The substantive results were the same as those obtained using survival models. Using logistic regressions models this way does not account for possible correlation between recurrent ADRs, Additionally, exposure time is not taken into account in the logistics regression models, in contrast to time-to-event models described in the preceding sections.

## Discussions

This study has shown the utility of using correlated survival models to the analysis of recurrent adverse drug reactions events to account for possible dependence in the events. The models have been applied to a very important data based on structured surveillance of HIV patients on antiretroviral treatment in South Africa. The country has the largest number of people living with the HIV virus and on ART in the world. Age, gender, ARV regimen type, period of initiation of ART were found to be associated with the timing of HIV/AIDS drug related adverse reactions regardless of the analysis model used, though gender was not statistically significant. The highly prevalent ADRs included neuropathy, rash, cough and lipodystrophy, comparable with results found in Nwokike [14]. As in Reddenna et al. [6], neuropathy was the most prevalent adverse drug reaction condition.

Patients older that ages 38 years experienced significantly higher recurrence of ADRs compared to patients aged 30 years and less, which supports the findings previous studies [7,19]. Gender differentials were also found with females having higher risks of ADRs than males. Though not statistically significant, the finding of gender differences in experiencing ARV-related ADRs has been observed in other African settings [18,19,27].

Patients taking AZT + 3TC + NVP combination had higher rates of ADRs compared to patients on d4T + 3TC + EFZ. It has also been found that patients taking d4T + 3TC + NVP experienced higher rates of ADRs compared to patients taking d4T + 3TC + EFZ. These findings confirm previous ARV-related ADR data in Spaulding et al. [28] and Webster et al. [29]. Due to high rates of ARV-related ADRs, South Africa patients are now using Tenofovir (TDF) containing regimen as a first line ARV treatment. Depending on the prognosis, AZT + 3TC + NVP and then d4T + 3TC + EFZ/NVP are offered [30]. More pharmacovigilance studies are needed to compare ADRs among patients on TDF containing regimen with patients on AZT + 3TC + NVP, a regimen found with lower ADR rates in this study. Patients who started ART after 2009 had a lesser rate of occurrence of ADRs; this may be due better care and management of HIV treatment.

This study showing high rates of ARV-related adverse drug reactions has highlighted the added morbidity among HIV patients taking ARV treatment. As indicated in Mehta et al. [21] in contract to other ADRs, almost all ARV-related ADRs are often inevitable and unpredictable, which makes treatment of these ADRs problematic. This creates extra cost burden to most public health systems that are stretched over many important health problems. In terms of policy implication, a better understanding, timely and proactive pharmacovigilance surveillance and reporting, especially of problematic regimen and patients subgroups, of ARV-related ADRs is advisable [4].

### Limitations of the study

The findings from this study should be interpreted within caution. The surveillance data that was used did not have grading of the severity of ADRs. Some studies have graded the ADRs using the World Health Organization grading or the Hartwig scale to determine the level of severity to patients’ morbidity [10,19,31,32]. Not all the data in the database were used as some patients were registered into the surveillance system study long after they had already initiated on ART. Thus, information on ADRs prior to being enrolled into the study was missing; using these patients in the analyses would have biased timing of first ADR. Other relevant covariates including CD4 count, viral load and body mass index were not used as these data had a high rate of missing values.

Further limitation of the analyses in this study was the lack of adherence data, which has been found to affect the rate of ADRs due to inadequate treatment uptake [33]. There were very few patients in the study who were started on ARTs based on the 2010 South African guidelines. These patients were included under “others” category, therefore the Tenofovir (TDF) containing combination was not individually assessed in the analysis. Lastly, several factors that may predispose patients to adverse reactions of antiretroviral medications including non-antiretroviral co-administered medication and treatment; alcoholism and viral hepatitis co-infection were also not explored [34-36].

Regarding statistical models for recurrent events data analysis, other models that could have been used include Anderson and Gill [37] and conditional models of Prentice, William and Peterson [38] instead of the WLW marginal model. The specification of the frailty model that was used for analysis in this paper could be improved by included time dependant random effects [22]. In terms of missing data, imputation methods could have been used on the variables with missing values and included in the analysis.

## Conclusions

This study has shown differential effects of patient’s age and antiretroviral regimen on the risks of adverse drug reactions in HIV-infection populations. It has highlighted the important of optimal drug selection and monitoring of vulnerable patients in mitigating the clinical severity of the adverse reactions. Further analyses using much larger and more complete data from different settings would be needed to solidify the findings in this study. Using correlated survival models is encouraged; however most of these data are observational in nature, which makes ascertaining casualty and associations problematic. Thus, to fully understand the determinants and dynamics of ADRs in HIV patients on ART, statistical methods including matching, propensity scores and instrumental variables techniques and where it is ethically possible, pharmacovigilance clinical trials should be encouraged. ADRs in HIV patients on ARV treatment are a huge public health problem in the free-delivery of ARV, especially in the high HIV epidemic countries of the sub-Saharan, where health resources are very limited.

### Ethics approval

The project proposal was submitted to and approved by the Medunsa Research and Ethics Committee at the University of Limpopo in 2006 (Project number MP119/2006).
